# Dose–response association between maternal pre-pregnancy bodyweight and gestational diabetes mellitus following ART treatment: a population-based cohort study

**DOI:** 10.1186/s12958-022-00964-9

**Published:** 2022-06-22

**Authors:** Yiquan Xiong, Jing Wang, Yana Qi, Chunrong Liu, Mingxi Li, Guanhua Yao, Wei Sun, Yongyao Qian, Lishan Ye, Hui Liu, Qiushi Xu, Kang Zou, Jing Tan, Xin Sun

**Affiliations:** 1 Chinese Evidence-Based Medicine Center, West China Hospital, Sichuan University, Chengdu, 610041 China; 2 NMPA Key Laboratory for Real World Data Research and Evaluation in Hainan, Chengdu, 610041 China; 3 Sichuan Center of Technology Innovation for Real World Data, Chengdu, 610041 China; 4 Xiamen Health Commission, Xiamen, 361000 China; 5 Xiamen Health and Medical Big Data Center, Xiamen, 361008 China

**Keywords:** Gestational diabetes mellitus, BMI, Dose–response analysis, Assisted reproductive technology, Population-based study

## Abstract

**Background:**

The impact of maternal pre-pregnancy bodyweight on gestational diabetes mellitus (GDM) following assisted reproductive technology (ART) treatment has been insufficiently investigated. The aim of this study was to investigate the association between maternal pre-pregnancy bodyweight and GDM following ART.

**Methods:**

From January 2014 to March 2019, this population-based retrospective cohort study included pregnancies achieved by ART treatment in a pregnancy registration database in China. Multivariate regression analysis and restricted cubic splines were used to explore the association between bodyweight and GDM.

**Results:**

A total of 6,598 pregnancies were included. The incidence of GDM was 26.0% (1715/6598). A total of 868 (13.2%) pregnant women were underweight, 665 (10.8%) were overweight, and 145 (2.20%) were obesity. We found a linear dose–response relation between maternal body mass index and GDM by restricted cubic splines, where one unit body mass index increase was associated with the 15% elevated risk of GDM (adjusted odds ratio [OR] 1.15, 95% CI 1.08–1.22). Compared to the normal weight group, maternal underweight was associated with lower risk of GDM (adjusted OR 0.68, 95% CI 0.57–0.82), while increased risk was found for overweight (adjusted OR 1.54 95% CI 1.29–1.84) and obesity (adjusted OR 1.74, 95% CI 1.23–2.47).

**Conclusions:**

Our study found a linear dose–effect relationship between pre-pregnancy bodyweight and GDM following ART treatment. The findings in this study support the clinical recommendation of advising women with overweight or obesity to lose weight prior to ART treatment.

**Supplementary Information:**

The online version contains supplementary material available at 10.1186/s12958-022-00964-9.

## Background

Gestational diabetes mellitus (GDM) is the most common pregnancy complications [1]. It was estimated that about 13.4% (16.9 million) of 2021 live births worldwide were affected by GDM [2]. This condition is associated with a well-documented short- and long-term health outcomes for mothers and their offspring, including preterm birth, low birth weight, neonatal unit admission, obesity in offspring, and type 2 diabetes after delivery [3–6]. Prior study suggested pre-pregnancy bodyweight was the critical risk factor for GDM in the general population [7, 8], given its rapid increase of overweight and obesity in prevalence globally [9, 10], it has been put under the spotlight in recent studies [8, 11].

Endocrine abnormalities are the main cause of infertility as result of the ovulation disorders [12]. Pre-pregnancy bodyweight, as a critical indicator of endocrine regulation, has a significant impact on pregnancy outcomes following assisted reproductive technology (ART). With excess body weight (i.e., overweight or obesity) on the rise in the general population[13], it has also become more prevalent among women undergoing ART treatment (e.g., in vitro fertilization [IVF] and intracytoplasmic sperm injection [ICSI]) [14]. Whether weight loss should be performed prior to ART has been a concern in reproductive clinical practice [15–17]. In a randomized controlled trial, Sim et al*.* reported that weight loss intervention prior to ART was associated with a significant improvement in pregnancy rates (48% *vs.* 14%, *P* = 0.01) and live births (44% *vs.* 14%, *P* = 0.02) in women with obesity [16]. Previous study shown that pregnant women with overweight and obesity were significantly associated increase the risk of GDM in spontaneous conceptions [7, 11]. Furthermore, a linear dose–response relationship between pre-pregnancy bodyweight and risk of GDM has been established [11]. However, it remains unknown whether there is a dose–response relationship between pre-pregnancy bodyweight and risk of GDM among ART population [18, 19]. There were two prior studies shown that both pregnant women with overweight and obesity was associated with increased risk of GDM following ART [18, 19], but merely examined the association between categories of maternal BMI (e.g., overweight, obesity) and GDM following ART, and a dose–response relationship between maternal BMI and GDM following ART has yet to be established.

Considering the particularities of assisted conception (i.e., cause of subfertility and ART procedure) and higher incidence of GDM following ART [20], we aimed to evaluate if the impact of maternal bodyweight on GDM incidence following ART was similar to that describe in spontaneous conceptions, since this subject has been insufficiently investigated [18]. In order to provide evidence for future interventions in maternal body weight before ART treatment, we conducted a dose–response analysis to explore the impact of maternal pre-pregnancy body weight on GDM following ART treatment, using data from REPRESENT [21].

## Methods

This population-based retrospective cohort study was approved by the Ethics Committee of West China Hospital (2019–825) in Sichuan, China and registered at clinicaltrials.gov (NCT04222621).

### Data sources

This study was conducted using data from REPRESENT, a population-based pregnancy registration database in China, which is described elsewhere [21]. Briefly, REPRESENT collected healthcare data from pregnant women and their offspring across all maternity departments in Xiamen city, Fujian Province, China, which has a permanent population of more than 4 million. REPRESENT establishment was primarily based on the Maternal and Child Health Management Platform and linked to three other platforms in Xiamen (Residents Healthcare Management Platform, Primary Healthcare Management Platform, and Electronic Healthcare Records Platform). A substantial volume of variables, including demographic characteristics, pregnancy co-morbidities, gestational complications, and pregnancy outcomes, were recorded by clinicians in the REPRESENT. While building the REPRESENT database, 1000 cases were randomly selected for verification, and the results showed that these cases were 100% complete and consistent with raw data. In January 2014, a screening checklist for pregnancy risk was uniformly implemented in REPRESENT, including conception mode (i.e., ART or natural).

### Participant selection

The pregnancies achieved by ART treatment (i.e., IVF and ICSI) were initially included in this study. Pregnancies that were > 20 weeks of gestation at the first antenatal visit or were not delivered in Xiamen were excluded in our analyses. Pregnant women with chronic hypertension, pregestational diabetes mellitus, psychosis, thalassemia, epilepsy, hyperthyroidism, hypothyroidism, were excluded, as were those who were infected with human immunodeficiency virus or syphilis or had hepatitis B e antigen seropositivity.

### Exposure definition

Maternal pre-pregnancy bodyweight was self-reported by pregnant women and recorded in the database by clinicians at the first antenatal visit. Bodyweight status was represented by body mass index (BMI), which was calculated by dividing the weight (kg) by squared height (m^2^). According to the standard in China [22], a BMI between 18.5 and 23.99 kg/m^2^ was considered as normal weight, while BMI < 18.5 kg/m^2^, between 24.0 and 27.99 kg/m^2^, and > 28.0 kg/m^2^ were considered as underweight, overweight and obesity, respectively.

### Outcome definition

The diagnosis of GDM in this study was based on 75-g oral glucose tolerance test at 24–28 weeks of gestation, with threshold values for fasting 5.1 mmol/L, 1 h 10.0 mmol/L, and 2 h 8.5 mmol/L, respectively, followed the criteria proposed by International Association of the Diabetes and Pregnancy Study Groups criteria (IADPSGC) [23], and endorsed by the obstetrics and gynecology branch of the Chinese Medical Association [24].

### Statistical analyses

To assess the risk of GDM in subjects with abnormal bodyweight, crude ORs with corresponding 95% confidence intervals (CIs) were calculated in univariate analysis. In multivariate analysis, four potential confounding factors including maternal age, education level (< 9 years, 10–12 years, or ≥ 13 years), parity (nulliparity or multiparity), and multiple gestations (singleton or multiples) were controlled, and the adjusted ORs and 95% CIs were calculated. Further, we used restricted cubic splines to flexibly model the dose–response relationship between maternal pre-pregnancy BMI and GDM. The spline models included the four potential confounders listed above. In order to have enough observations in between the knots to fit each polynomial [25], according to routinely approach, three knots at the 10th, 50th, and 90th centiles were used to examine potential non-linear associations [26]. These associations were subjected to likelihood ratio tests comparing the model with only a linear term against one with linear and cubic spline terms [27, 28]. A *P* value for non-linearity test (*P*_*n*_) < 0.05 was considered as a nonlinear dose–response relationship. Two subgroup analyses were conducted by stratifying the participants by maternal age (< 35 vs. ≥ 35 years old) and multiple gestations (singleton vs. multiples).

Additionally, we conducted a sensitivity analysis by redefining bodyweight classification according to World Health Organization (WHO) standard: underweight (BMI < 18.5 kg/m^2^), normal weight (BMI 18.5–24.99 kg/m^2^), overweight (BMI 25.0–29.99 kg/m^2^), and obesity (BMI ≥ 30.0 kg/m^2^). Because there were only 22 women with BMI ≥ 30.0 kg/m^2^, these women with obesity were omitted in sensitivity analysis.

Categorical variables are expressed as frequencies and percentages. Pearson’s χ^2^ or Fisher’s exact tests were used to test comparison between groups of categorical variables. The distribution of continuous variables was tested by performing the Kolmogorov–Smirnov test, and mean ± SD values were used to describe variables with a normal distribution; otherwise, median values and interquartile ranges (IQRs) were used. All statistical analyses were performed with R v3.6.1 (R Project for Statistical Computing, Austria, Vienna). *P* < 0.05 (two-tailed) was considered significant.

## Results

### Population characteristics

**Fig. 1:**
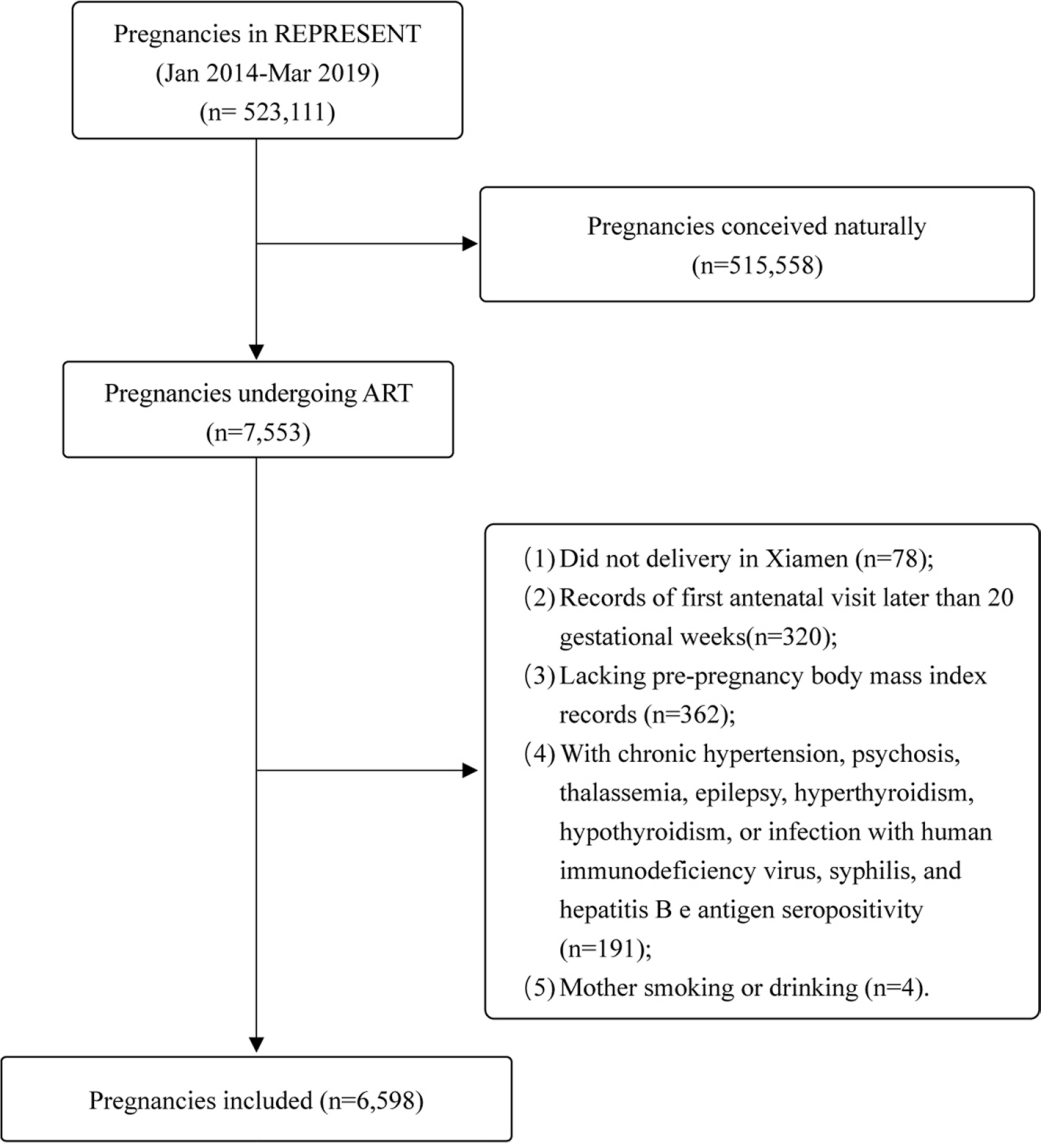
Flow chart of pregnancies included in this study. ART, assisted reproductive technology; HIV, human immunodeficiency virus

From January 2014 to March 2019, REPRESENT accumulated data from 523,111 pregnancies. Among these pregnancies, 7,553 pregnancies following ART treatment, and 6,598 pregnancies were included in our analyses after applying the exclusion criteria (Fig. 1). In this study, there were 868 (13.16%) women with pre-pregnancy underweight, 4920 (74.57%) with normal weight, 665 (10.08%) women with overweight, and 145 (2.20%) women with obesity (Table 1). The median maternal age was 31 (IQR: 28–41), and 20.86% of pregnant women were ≥ 35 years old (Table 1). Among the four BMI groups, there were significant differences regarding maternal age, education level, and parity (Table 1). There were no significant differences in location, permanent population, or multiple gestations.

**Table 1:** Maternal demographic and obstetric characteristics

		Total	BMI < 18.5 kg/m^2^ (n = 868)	BMI:18.5–24 kg/m^2^ (n = 4920)	BMI: 24–28 kg/m^2^ (n = 665)	BMI > = 28 kg/m^2^ (n = 145)	Chi-square value	*P* value
Maternal age		6596	868	4918	665	145		
	< 25	167 (2.53)	27 (3.11)	122 (2.48)	13 (1.95)	5 (3.45)	108.9	< 0.001
	25–30	2116 (32.08)	361 (41.59)	1535 (31.21)	178 (26.77)	42 (28.97)		
	30–35	2937 (44.53)	372 (42.86)	2242 (45.59)	264 (39.70)	59 (40.69)		
	> = 35	1376 (20.86)	108 (12.44)	1019 (20.72)	210 (31.58)	39 (26.90)		
Education level (years)		6483	855	4840	646	142		
	< = 9	1722 (26.56)	211 (24.68)	1234 (25.50)	221 (34.21)	56 (39.44)	54.79	< 0.001
	10–12	1334 (20.58)	175 (20.47)	980 (20.25)	159 (24.61)	20 (14.08)		
	> 12	3427 (52.86)	469 (54.85)	2626 (54.26)	266 (41.18)	66 (46.48)		
Location		6598	868	4920	665	145		
	city or town	5960 (90.33)	784 (90.32)	4446 (90.37)	599 (90.08)	131 (90.34)	0.06	0.99
	rural area	638 (9.67)	84 (9.68)	474 (9.63)	66 (9.92)	14 (9.66)		
Permanent population		6593	868	4915	665	145		
	No	4797 (72.76)	613 (70.62)	3591 (73.06)	482 (72.48)	111 (76.55)	3.31	0.35
	Yes	1796 (27.24)	255 (29.38)	1324 (26.94)	183 (27.52)	34 (23.45)		
Parity		6598	868	4920	665	145		
	Nullipara	5339 (80.92)	757 (87.21)	3965 (80.59)	501 (75.34)	116 (80.00)	36.1	< 0.001
	Multipara	1259 (19.08)	111 (12.79)	955 (19.41)	164 (24.66)	29 (20.00)		
Multiple gestations		6598	868	4920	665	145		
	Singleton	4806 (72.84)	615 (70.85)	3592 (73.01)	487 (73.23)	112 (77.24)	3.28	0.35
	Multiple gestations	1792 (27.16)	253 (29.15)	1328 (26.99)	178 (26.77)	33 (22.76)		

Data are presented as n (%)

*BMI* body mass index (kg/m^2^)

### The incidence of GDM following ART

The overall incidence of GDM was 25.99% (1715/6598). The incidence in pregnant women older than 35 years old was significantly higher than that in the < 35 group (35% *vs.* 24%, χ^2^ = 37.35, *P* < 0.001) (Table 2). There was no significant difference between singleton and multiple gestations (26% *vs.* 25%, χ^2^ = 0.22, *P* = 0.64).

**Table 2:** Overall and subgroup analyses of the association between maternal pre-pregnancy bodyweight and gestational diabetes mellitus

		Total	BMI:18.5–24 kg/m2	BMI < 18.5 kg/m^2^			BMI: 24–28 kg/m^2^			BMI > = 28 kg/m^2^		
				n (%)	cOR	aOR (95% CI)	n (%)	cOR	aOR (95% CI)	n (%)	cOR	aOR (95% CI)
**Overall analysis**		1715 (25.99)	1264 (25.69)	158 (18.20)	0.64 (0.54–0.77)	0.68 (0.57–0.82)	238 (35.79)	1.61 (1.36–1.91)	1.54 (1.29–1.84)	55 (37.93)	1.77 (1.26–2.49)	1.74 (1.23–2.47)
**maternal age**^a^												
	< 35	1239 (23.74)	918 (23.54)	138 (18.16)	0.72 (0.59–0.88)	0.72 (0.59–0.88)	150 (32.97)	1.60 (1.30–1.97)	1.62 (1.31–2.00)	33 (31.13)	1.47 (0.97–2.23)	1.47 (0.96–2.25)
	> = 35	476 (34.59)	346 (33.95)	20 (18.52)	0.44 (0.27–0.73)	0.43 (0.26–0.71)	88 (41.90)	1.40 (1.04–1.90)	1.40 (1.03–1.92)	22 (56.41)	2.52 (1.32–4.80)	2.70 (1.41–5.19)
**Multiple gestations**^b^												
	Singleton	1259 (26.20)	942 (26.22)	96 (15.61)	0.52 (0.41–0.65)	0.56 (0.44–0.70)	176 (36.14)	1.59 (1.30–1.94)	1.55 (1.26–1.91)	45 (40.18)	1.89 (1.29–2.78)	1.89 (1.28–2.80)
	Multiple gestations	456 (25.45)	322 (24.25)	62 (24.51)	1.01 (0.74–1.39)	1.05 (0.76–1.45)	62 (34.83)	1.67 (1.20–2.33)	1.54 (1.10–2.18)	10 (30.30)	1.36 (0.64–2.88)	1.28 (0.59–2.76)

Data are presented as n (%); a, the incidence of GDM was significantly difference between the two maternal age groups (χ^2^ = 37.35, *P* < 0.001); b, the incidence of GDM was not significantly difference between the two gestations groups (χ.^2^ = 0.22, *P* = 0.64)

*BMI* body mass index (kg/m^2^), *cOR* crude odds ratio, *aOR* adjusted odds ratio

### The impact of maternal pre-pregnancy bodyweight on GDM

Multivariate analysis indicated that compared to normal weight, women with pre-pregnancy underweight had decreased risk of GDM (18.20% vs. 25.69%, adjusted OR 0.68, 95% CI 0.57–0.82). Conversely, women with pre-pregnancy overweight (adjusted OR 1.54, 95% CI 1.29–1.84) and obesity (adjusted OR 1.74, 95% CI 1.23–2.47) had increased risk of GDM (Table 2). For both age subgroups, maternal underweight decreased the risk of GDM, while overweight and obesity increased the risk (Table 2). The results for singleton gestations were consistent with the overall analyses, but the association between underweight, obesity and GDM was not significant for multiple gestations (Table 2). Sensitivity analysis according to the WHO BMI classification criteria also showed that underweight status decreased the risk of GDM (adjusted OR 0.65, 95% CI 0.54–0.79), while it was increased in overweight subjects (adjusted OR 1.40, 95% CI 1.13–1.73).

**Fig. 2:**
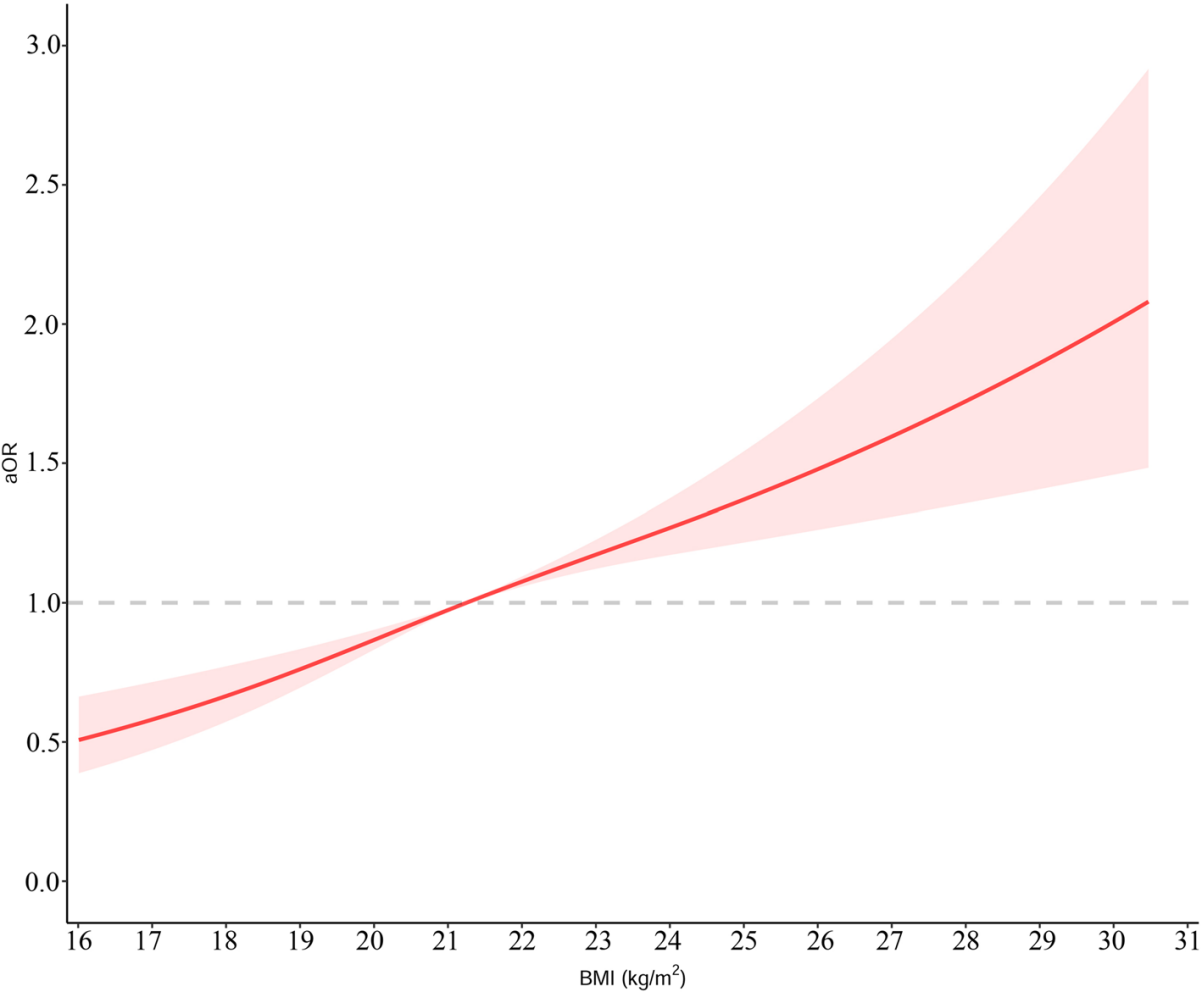
The dose‐response relationship of maternal pre-pregnancy body mass index (BMI, kg/m^2^) and risk of GDM based on the linear model. The solid line represents the fitted linear trend, and shaded areas represents the 95% confidence interval. aOR, adjusted odds ratio

The restricted cubic splines revealed a linear dose–response relationship between maternal pre-pregnancy BMI and GDM (*P* value for dose–response relation test [*Pd*] < 0.001, *P*_*n*_ = 0.19) (Fig. 2). The results indicated that increased maternal BMI was associated with a higher risk of GDM. The adjusted OR of each 1-unit increase in BMI for GDM was 1.15 (95% CI 1.08–1.22) and that of each 5-unit increase was 1.98 (95% CI 1.45–2.66). When maternal BMI decreased from 18.5 to 16.0, the corresponding risk of GDM went from 0.71 (95% CI 0.63–0.80) to 0.51 (95% CI 0.39–0.66) (Table S1). Conversely, when maternal BMI rose from 24.0 to 30.5, the corresponding risk of GDM increased from 1.27 (95% CI 1.17–1.38) to 2.08 (95% CI 1.48–2.92) (Table S1).

**Fig. 3:**
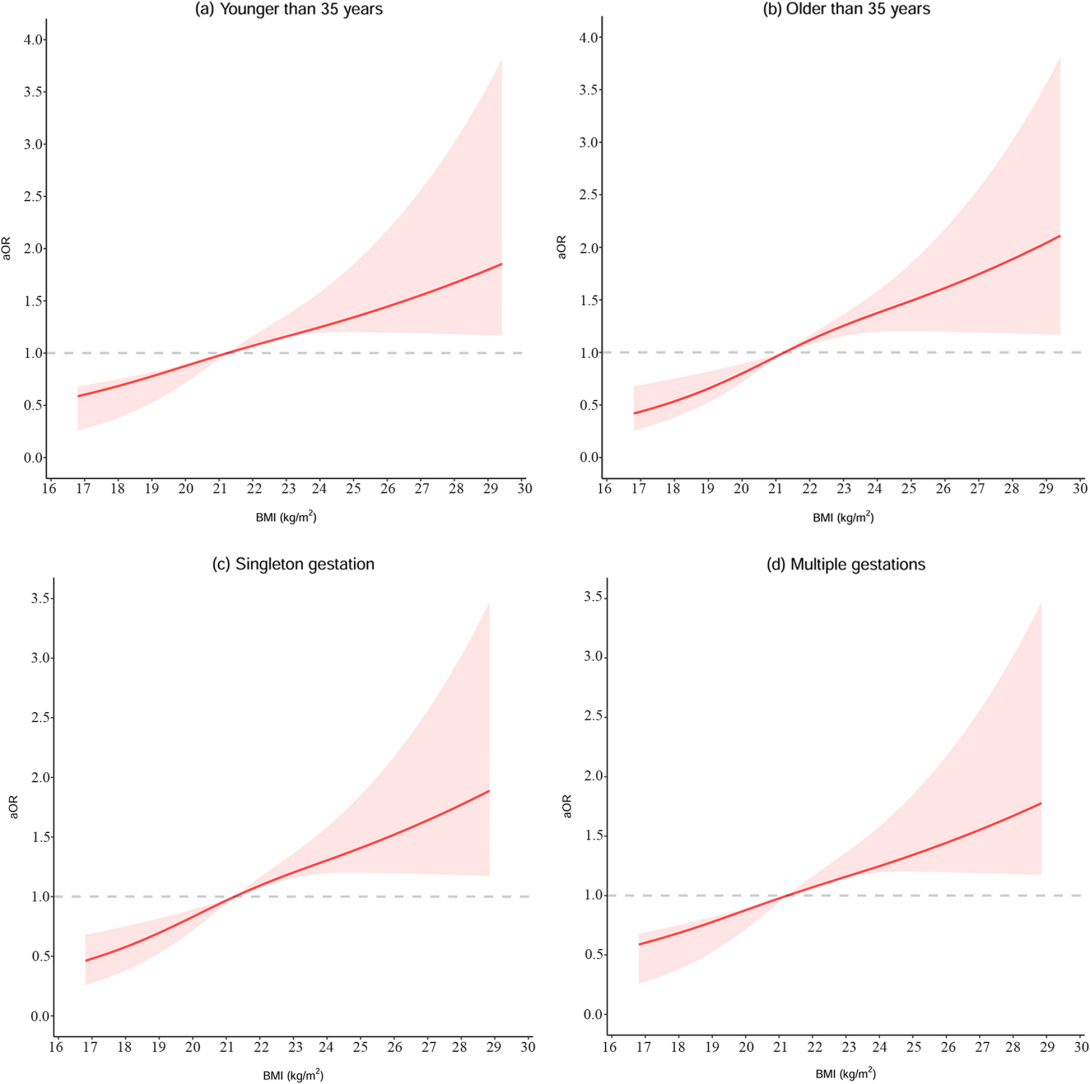
Dose–response relationship between maternal pre-pregnancy BMI (kg/m^2^) and gestational diabetes mellitus in subgroups: (**a**) maternal age younger than 35 years old, (**b**) maternal age older than 35 years old, (**c**) singleton gestations, (**d**) multiple gestations. The solid line represents the fitted linear trend, and shaded areas represents the 95% confidence interval. aOR, adjusted odds ratio

Subgroup analyses indicated a significant linear dose–response relations between maternal pre-pregnancy BMI and GDM in subjects younger than 35 years old (*P*_*d*_ < 0.001, *P*_*n*_ = 0.29), older than 35 years old (*P*_*d*_ < 0.001, *P*_*n*_ = 0.18), and those with multiple gestations (*P*_*d*_ = 0.03, *P*_*n*_ = 0.67) (Fig. 3). The respective adjusted ORs of each 1-unit increase in BMI for GDM in subjects younger than 35 years old was 1.14 (95% CI 1.06–1.22), in older than 35 years old was 1.23 (95% CI 1.09–1.38), and in multiple gestations was 1.03 (95% CI 0.93–1.16). A non-linear dose–response relationship was observed in singleton gestations (*P*_*d*_ < 0.001, *P*_*n*_ = 0.04) (Fig. 3).

## Discussion

After adjusting for confounding factors, maternal underweight (adjusted OR 0.68) was associated with decreased risk of GDM following ART compared to women with normal weight, while overweight (adjusted OR 1.54) and obesity (adjusted OR 1.74) were associated with increased risk. In addition, there is a linear increase in the risk of GDM following ART with higher maternal pre-pregnancy BMI. For every 1-unit BMI increase, the risk of GDM increased by 15% (adjusted OR 1.15).

Few studies have investigated the impact of maternal pre-pregnancy bodyweight on GDM following ART. Using the WHO BMI classification criteria, we found a significant impact of maternal bodyweight on GDM, with adjusted OR of 0.65 (95% CI 0.54–0.79) for underweight and 1.40 (95% CI 1.13–1.73) for overweight, which was consistent with a previous Slovenian study [18]. Lucovnik et al. reported that when compared with normal weight, the adjusted OR for GDM in IVF singleton pregnancies was 0.4 (95% CI 0.2–0.9) in underweight group, 2.2 (95% CI 1.7–2.7) in overweight group, and 5.4 (95% CI 4.3–6.7) in obesity group [18]. In another study, Frankenthal et al*.* reported that the incidence of GDM following ART was 8.4% in normal weight group, 17.4% in overweight group, and 22.0% in obesity group, but the incidence in underweight group was 13.1% [19].

On the other hand, the impact of maternal bodyweight on GDM in spontaneous conceptions has been widely investigated [7, 8, 11, 24, 27]. An individual participant data meta-analysis shown that the pooled risk for GDM was 0.66 in maternal underweight group, 2.22 in overweight group, 3.97 in obese grade 1 group, 5.85 in obese grade 2 group, and 7.59 in obese grade 3 group [7]. In addition, based on 10 studies, Najafi et al*.* reported the risk of GDM increases by 14% (pooled adjusted OR 1.14, 95% CI 1.10‐1.19) with an increase in one unit of BMI after adjusted confounders [11]. In our study, similar linear dose–response association, the risk of GDM following ART increases by 15% (adjusted OR 1.15, 95% CI 1.08–1.22) with an increase in one unit of BMI, was observed. These results suggested that the impact of maternal bodyweight on GDM in assisted conceptions probably similar to that in spontaneous conceptions.

For women with overweight or obesity, whether weight loss should be performed prior to ART has been a popular topic of concern. Several studies indicated that weight loss prior to ART has shown benefits in increasing pregnancy rate or live birth rate in women with overweight or obesity [29]. On the other hand, whether weight loss has benefits in decreasing the risk of pregnancy complications (e.g., GDM) was unknown. Reducing the risk of GDM following ART will benefit a range of short- and long-term health implications for mothers and their offspring [3–6]. In the present study, compared with normal weight, maternal excess maternal bodyweight was associated with increased risk of GDM following ART and a dose–response relationship was observed. These findings support the clinical recommendation of advising women with overweight or obesity to lose weight prior to ART.

It was reported that maternal advanced age was associated with increased risk of GDM following ART [28, 30]. In the present study, the incidence of GDM following ART in subjects older than 35 years old was higher than that in pregnant women younger than 35 years old (34.59% *vs.* 23.74%, *P* < 0.001). The age-stratified analysis showed linear dose–response relationships between maternal pre-pregnancy bodyweight and GDM in both age groups. After adjusting for confounders, the risk of GDM increased by 23% and 14% with a 1-unit increase in BMI in woman older and younger than 35 years old, respectively. This suggested that, women with overweight or obesity, the benefit of weight intervention before starting ART procedure in women older than 35 years old probably greater than that in women younger than 35 years old. In addition, during pregnancy, more comprehensive antenatal care should be performed in excess bodyweight women older than 35 years old.

In conception achieved by ART, multiple gestations usually increase the risk of adverse pregnancy outcomes [31, 32]. A previous study indicated that GDM following ART was more common in multiple gestations compared to singleton with OR of 1.30 (95% CI 1.10–1.54) [33]. In the present study, the incidence rates of GDM following ART were similar in singleton and multiple gestations (26.20% *vs.* 25.45%, *P* = 0.64). Both types of gestations also showed significant dose–response relationships between maternal pre-pregnancy bodyweight and GDM. In specific, the risk of BMI at 25.0 kg/m^2^ and 28.0 kg/m^2^ for GDM was 1.41 and 1.78 in singleton, and those in multiple gestations was 1.28 and 1.56. The impact of maternal pre-pregnancy bodyweight on GDM following ART in singleton seems similar with that in multiple gestations.

In our study, less than 20% of women presented excessive weight (overweight or obesity), which is in contrast with the current pattern of BMI distribution described in most developed countries [13]. For example, there were only 2.8% of pregnant women following ART with underweight, on the contrary, 42.1% with overweight or obesity in a national study in USA [34]. Since the limited number of pregnant women with obesity, the findings regarding the risk of obesity for GDM should be interpreted with caution. The risk of obesity for GDM is definitely higher than that in overweight (adjusted OR 1.40, 95% CI 1.13–1.73), such as adjusted OR of 5.4 (95% CI 4.3–6.7) reported in a previous study [18]. The dose–effect association between maternal obesity and risk of GDM following ART should need further research.

To the best of our knowledge, this is the first study to evaluate the dose–response relationship between maternal pre-pregnancy bodyweight and GDM in population underwent assisted conceptions. There were several strengths in this investigation. First, this was a population-based study that included almost all pregnancies achieved by ART treatment in Xiamen City, which enhanced the generalizability of the results. Second, in addition to conventional analysis that divided BMI into several categories, we used restricted cubic splines to evaluate the dose–response relationships between maternal pre-pregnancy bodyweight and GDM following ART, thus providing the precise evidence for pre-pregnancy weight loss before ART admission. In addition, by the multiple statistical strategies, including multivariate regression, subgroup analyses, and sensitivity analyses, we drew the robustness of the findings. However, the present study had three main limitations. First, the impact of maternal pre-pregnancy bodyweight and different ART methods (i.e., IVF or ICSI) remains unclear. Further investigation is needed to analyze the effects of obesity and IVF *vs.* ICSI on GDM. Second, because of the retrospective study design, some confounding factors (e.g., family history of diabetes mellitus) were not included in the analysis. Third, although this is a population-based study, the proportion of women with overweight or obesity was relatively low compared with that in developed countries, which may limit the generalizability of our findings.

## Conclusions

We found a dose–effect association between maternal pre-pregnancy bodyweight and risk of GDM following ART. There was a linear increase in GDM risk with higher maternal pre-pregnancy BMI, which adds to the evidence for supporting weight intervention before starting ART procedure treatment for women with excess pre-pregnancy bodyweight.

## Supplementary Information

**Additional file 1: Table S1.** the risk ofdifferent maternal BMI for gestational diabetesmellitus.

## Data Availability

The datasets analyzed in the current study are not publicly available, but are available from the corresponding author on reasonable request.

## Abbreviations

ART: Assisted reproductive technology
BMI: Body mass index
CI: Confidence interval
GDM: Gestational diabetes mellitus
ICSI: Intracytoplasmic sperm injection
IVF: In vitro fertilization
OR: Odds ratio
REPRESENT: A population-based pregnancy registration database in China
WHO: World Health Organization
IQRs: Interquartile ranges

## References

[1] American Diabetes A (2018). 2. Classification and Diagnosis of Diabetes: Standards of Medical Care in Diabetes-2018. Diabetes Care.

[2] International Diabetes Federation, IDF Diabetes Atlas 2019 (9th edn). Available athttps://diabetesatlas.org/en/resources/. Accessed 1 Aug 2021. 35914061

[3] Billionnet C, Mitanchez D, Weill A, Nizard J, Alla F, Hartemann A, Jacqueminet S (2017). Gestational diabetes and adverse perinatal outcomes from 716,152 births in France in 2012. Diabetologia.

[4] Wendland EM, Torloni MR, Falavigna M, Trujillo J, Dode MA, Campos MA, Duncan BB, Schmidt MI (2012). Gestational diabetes and pregnancy outcomes–a systematic review of the World Health Organization (WHO) and the International Association of Diabetes in Pregnancy Study Groups (IADPSG) diagnostic criteria. BMC Pregnancy Childbirth.

[5] Johns EC, Denison FC, Norman JE, Reynolds RM (2018). Gestational Diabetes Mellitus: Mechanisms, Treatment, and Complications. Trends Endocrinol Metab.

[6] Buchanan TA, Xiang AH, Page KA (2012). Gestational diabetes mellitus: risks and management during and after pregnancy. Nat Rev Endocrinol.

[7] Santos S, Voerman E, Amiano P, Barros H, Beilin LJ, Bergstrom A, Charles MA, Chatzi L, Chevrier C, Chrousos GP (2019). Impact of maternal body mass index and gestational weight gain on pregnancy complications: an individual participant data meta-analysis of European North American and Australian cohorts. BJOG.

[8] Torloni MR, Betran AP, Horta BL, Nakamura MU, Atallah AN, Moron AF, Valente O (2009). Prepregnancy BMI and the risk of gestational diabetes: a systematic review of the literature with meta-analysis. Obes Rev.

[9] Ogden CL, Carroll MD, Kit BK, Flegal KM (2014). Prevalence of childhood and adult obesity in the United States, 2011–2012. JAMA.

[10] Flegal KM, Carroll MD, Kit BK, Ogden CL (2012). Prevalence of obesity and trends in the distribution of body mass index among US adults, 1999–2010. JAMA.

[11] Najafi F, Hasani J, Izadi N, Hashemi-Nazari SS, Namvar Z, Mohammadi S, Sadeghi M (2019). The effect of prepregnancy body mass index on the risk of gestational diabetes mellitus: A systematic review and dose-response meta-analysis. Obes Rev.

[12] Carson SA, Kallen AN (2021). Diagnosis and Management of Infertility: A Review. JAMA.

[13] WHO. Global Health Observatory (GHO) data. Available at:https://www.who.int/gho/ncd/risk_factors/overweight_text/en/. Accessed 7 Feb 2022.

[14] Sermondade N, Huberlant S, Bourhis-Lefebvre V, Arbo E, Gallot V, Colombani M, Freour T (2019). Female obesity is negatively associated with live birth rate following IVF: a systematic review and meta-analysis. Hum Reprod Update.

[15] Liu L, Wang H, Zhang Y, Niu J, Li Z, Tang R (2020). Effect of pregravid obesity on perinatal outcomes in singleton pregnancies following in vitro fertilization and the weight-loss goals to reduce the risks of poor pregnancy outcomes: A retrospective cohort study. PLoS ONE.

[16] Sim KA, Dezarnaulds GM, Denyer GS, Skilton MR, Caterson ID (2014). Weight loss improves reproductive outcomes in obese women undergoing fertility treatment: a randomized controlled trial. Clin Obes.

[17] Einarsson S, Bergh C, Friberg B, Pinborg A, Klajnbard A, Karlstrom PO, Kluge L, Larsson I, Loft A, Mikkelsen-Englund AL (2017). Weight reduction intervention for obese infertile women prior to IVF: a randomized controlled trial. Hum Reprod.

[18] Lucovnik M, Blickstein I, Mirkovic T, Verdenik I, Bricelj K, Vidmar Simic M, Tul N, Trojner Bregar A (2018). Effect of pre-gravid body mass index on outcomes of pregnancies following in vitro fertilization. J Assist Reprod Genet.

[19] Frankenthal D, Hirsh-Yechezkel G, Boyko V, Orvieto R, Ron-El R, Lerner-Geva L, Farhi A (2019). The effect of body mass index (BMI) and gestational weight gain on adverse obstetrical outcomes in pregnancies following assisted reproductive technology as compared to spontaneously conceived pregnancies. Obes Res Clin Pract.

[20] Bosdou JK, Anagnostis P, Goulis DG, Lainas GT, Tarlatzis BC, Grimbizis GF, Kolibianakis EM (2020). Risk of gestational diabetes mellitus in women achieving singleton pregnancy spontaneously or after ART: a systematic review and meta-analysis. Hum Reprod Update.

[21] Tan J, Xiong Y, Qi Y, Liu C, Huang S, Yao G, Sun W, Qian Y, Ye L, Xu Q (2021). Data Resource Profile: Xiamen registry of pregnant women and offspring (REPRESENT): a population-based, long-term follow-up database linking four major healthcare data platforms. Int J Epidemiol.

[22] Committee. HCAP: Healthy China Action (2019–2030). 2019. Available at: http://www.gov.cn/xinwen/2019-07/15/content_5409694.htm. Accessed 1 Aug 2021.

[23] Metzger BE, Gabbe SG, Persson B, Buchanan TA, Catalano PA, Damm P, Dyer AR, Leiva A (2010). International association of diabetes and pregnancy study groups recommendations on the diagnosis and classification of hyperglycemia in pregnancy. Diabetes Care.

[24] Yao D, Chang Q, Wu QJ, Gao SY, Zhao H, Liu YS, Jiang YT, Zhao YH (2020). Relationship between Maternal Central Obesity and the Risk of Gestational Diabetes Mellitus: A Systematic Review and Meta-Analysis of Cohort Studies. J Diabetes Res.

[25] Harrell (2010). Regression Modeling Strategies: with applications to linear models, logistic regression, and survival analysis.

[26] Durrleman S, Simon R (1989). Flexible regression models with cubic splines. Stat Med.

[27] Chu SY, Callaghan WM, Kim SY, Schmid CH, Lau J, England LJ, Dietz PM (2007). Maternal obesity and risk of gestational diabetes mellitus. Diabetes Care.

[28] Shiqiao H, Bei X, Yini Z, Lei J (2020). Risk factors of gestational diabetes mellitus during assisted reproductive technology procedures. Gynecol Endocrinol.

[29] Sim KA, Partridge SR, Sainsbury A (2014). Does weight loss in overweight or obese women improve fertility treatment outcomes? A systematic review. Obes Rev.

[30] Kouhkan A, Khamseh ME, Pirjani R, Moini A, Arabipoor A, Maroufizadeh S, Hosseini R, Baradaran HR (2018). Obstetric and perinatal outcomes of singleton pregnancies conceived via assisted reproductive technology complicated by gestational diabetes mellitus: a prospective cohort study. BMC Pregnancy Childbirth.

[31] Tan J, Qi YN, Zhang J, Wang W, Zhang GT, Zou K, Liu XH, Sun X (2019). The mediation effect of multiple gestations on the association between in vitro fertilisation and severe maternal morbidities: a retrospective cohort study. BMJ Open.

[32] Chen S, Du H, Liu J, Liu H, Li L, He Y (2020). Live birth rate and neonatal outcomes of different quantities and qualities of frozen transferred blastocyst in patients requiring whole embryo freezing stratified by age. BMC Pregnancy Childbirth.

[33] Luke B, Stern JE, Kotelchuck M, Declercq ER, Anderka M, Diop H (2016). Birth Outcomes by Infertility Treatment: Analyses of the Population-Based Cohort: Massachusetts Outcomes Study of Assisted Reproductive Technologies (MOSART). J Reprod Med.

[34] Kawwass JF, Kulkarni AD, Hipp HS, Crawford S, Kissin DM, Jamieson DJ (2016). Extremities of body mass index and their association with pregnancy outcomes in women undergoing in vitro fertilization in the United States. Fertil Steril.

