# Supplementary material for: Dose–response association between maternal pre-pregnancy bodyweight and gestational diabetes mellitus following ART treatment: a population-based cohort study
Source: Reprod Biol Endocrinol. 2022 Jun 22;20:92. doi: 10.1186/s12958-022-00964-9 (PMC9215080; doi:10.1186/s12958-022-00964-9)
Supplement: Supplementary file 1 — Additional file 1: Table S1. the risk ofdifferent maternal BMI for gestational diabetesmellitus. [file 12958_2022_964_MOESM1_ESM.docx]

Table S1 the risk of different maternal BMI for gestational diabetes mellitus.

| BMI | aOR (95% CI) |
| --- | --- |
| 16.00 | 0.51 (0.39-0.66) |
| 16.50 | 0.54 (0.43-0.69) |
| 17.00 | 0.58 (0.47-0.71) |
| 17.50 | 0.62 (0.52-0.74) |
| 18.00 | 0.67 (0.58-0.77) |
| 18.50 | 0.71 (0.63-0.80) |
| 19.00 | 0.76 (0.70-0.83) |
| 19.50 | 0.82 (0.77-0.87) |
| 20.00 | 0.86 (0.83-0.90) |
| 20.50 | 0.92 (0.90-0.94) |
| 21.00 | 0.98 (0.97-0.98) |
| 21.25 | 1 (reference) |
| 21.50 | 1.02 (1.02-1.03) |
| 22.00 | 1.08 (1.06-1.09) |
| 22.50 | 1.13 (1.09-1.16) |
| 23.00 | 1.17 (1.12-1.22) |
| 23.50 | 1.22 (1.15-1.30) |
| 24.00 | 1.27 (1.17-1.38) |
| 24.50 | 1.32 (1.19-1.45) |
| 25.00 | 1.37 (1.22-1.54) |
| 25.50 | 1.43 (1.24-1.64) |
| 26.00 | 1.48 (1.26-1.73) |
| 26.50 | 1.54 (1.29-1.84) |
| 27.00 | 1.60 (1.31-1.95) |
| 27.50 | 1.65 (1.33-2.06) |
| 28.00 | 1.72 (1.36-2.19) |
| 28.50 | 1.79 (1.38-2.32) |
| 29.00 | 1.86 (1.41-2.45) |
| 29.50 | 1.93 (1.43-2.60) |
| 30.00 | 2.01 (1.46-2.77) |
| 30.50 | 2.08 (1.48-2.92) |

BMI, body mass index (kg/m^2^); aOR, adjusted odds ratio.
